# Feasibility and Efficacy of Intra‐Arterial Administration of Mesenchymal Stem Cells in an Animal Model of Double Toxin‐Induced Multiple System Atrophy

**DOI:** 10.1002/sctm.16-0438

**Published:** 2017-03-13

**Authors:** Ha Na Kim, Dong Yeol Kim, Se Hee Oh, Hyung Sook Kim, Kyung Suk Kim, Phil Hyu Lee

**Affiliations:** ^1^ Department of Neurology, Yonsei University College of MedicineSeoulSouth Korea; ^2^ Severance Biomedical Science Institute, Yonsei UniversitySeoulSouth Korea; ^3^ Bioengineering InstituteCORESTEM Inc.GyeonggiSouth Korea

**Keywords:** Multiple system atrophy, Mesenchymal stem cells, Intra‐arterial injection, Feasibility, Efficacy

## Abstract

Multiple system atrophy (MSA) is a sporadic neurodegenerative disease of the central and autonomic nervous system. Because no drug treatment consistently benefits MSA patients, neuroprotective strategy using mesenchymal stem cells (MSCs) has a lot of concern for the management of MSA. In this study, we investigated the safety and efficacy of intra‐arterial administration of MSCs via internal carotid artery (ICA) in an animal model of MSA. The study was composed of feasibility test using a ×10 and ×50 of a standard dose of MSCs (4 × 10^7^ MSCs) and efficacy test using a ×0.2, ×2, and ×20 of the standard dose. An ultrasonic flow meter and magnetic resonance imaging (MRI) showed that no cerebral ischemic lesions with patent ICA blood flow was were observed in animals receiving a ×10 of the standard dose of MSCs. However, no MSA animals receiving a ×50 of the standard dose survived. In efficacy test, animals injected with a ×2 of the standard dose increased nigrostriatal neuronal survival relative to a ×0.2 or ×20 of the standard dose. MSA animals receiving MSCs at ×0.2 and ×2 concentrations of the standard dose exhibited a significant reduction in rotation behavior relative to ×20 of the standard dose of MSCs. Cerebral ischemic lesions on MRI were only observed in MSA animals receiving a ×20 of the standard dose. The present study revealed that if their concentration is appropriate, intra‐arterial injection of MSCs is safe and exerts a neuroprotective effect on striatal and nigral neurons with a coincidental improvement in motor behavior. Stem Cells Translational Medicine *2017;6:1424–1433*

**Significance Statement**

Multiple system atrophy (MSA) is a sporadic neurodegenerative disease of the central and autonomic nervous system. Because no drug treatment consistently benefits MSA patients, neuroprotective strategy using mesenchymal stem cells (MSCs) has a lot of concern for the management of MSA. Our study has provided important details that intra‐arterial administration of MSCs is feasible and efficient administration route, resulting in improvement of neuronal viability and behavioral performance in a double toxin‐induced MSA model. The present data may advance clinical applications of intra‐arterial route of MSC administration in patients with MSA.

## Introduction

Multiple system atrophy (MSA) is a sporadic neurodegenerative disease of the central and autonomic nervous system, and its clinical features include any combination of autonomic failure, parkinsonism, and pyramidal signs. Pathologically, MSA is regarded as a unique entity within the spectrum of oligodendrogliopathy, with extensive α‐synuclein‐positive glial cytoplasmic inclusions, and leading to striatonigral degeneration, olivopontocerebellar degeneration, astrogliosis, and microgliosis 1. Because of grave prognosis, many clinical studies have been conducted to modify disease progression in MSA, such as trials involving growth hormone, immunoglobulin, monoamine oxidase type B (MOB‐B) inhibitor, or rifampicine 2. However, these trials have failed to achieve neuroprotective efficacy.

Mesenchymal stem cells (MSCs) are multipotent stem cells present in adult bone marrow that are capable of differentiating into various cell types under appropriate conditions. Additionally, MSCs secrete various cytotropic factors including neurotrophic growth factors, chemokines, cytokines, and extracellular matrix protein, which in turn, exert neuroprotective effects 3, 4. Our previous studies show that MSCs have potent neuroprotective effects in animal models of parkinsonian disorders through modulation of neuroinflammation, inhibition of apoptotic cell death, increases in neurogenesis and neuronal differentiation, and enhancement of autophagy 5, 6, 7, 8. In addition, MSC exert a neuroprotective effect in animal model of MSA 9. Moreover, we recently demonstrated in investigator initiated trials that intra‐arterial administration of MSCs delays progression of neurological deficits assessed clinically and radiologically in patients with MSA 10.

Of several potential delivery routes, intra‐arterial administration of MSCs has received attention because this approach increases cell homing to the brain by circumventing the pulmonary circulation and thus leads to enhanced therapeutic outcomes 11, 12, 13. However, cerebral ischemia related to intra‐arterial cell infusion has been reported, although the complications related to intra‐arterial administration of stem cells seem to depend on the infusion technique, cell dose, and infusion velocity 14, 15. Therefore, a careful optimization of the intra‐arterial infusion procedures, especially with regard to cell dose is required before pharmacotherapeutic development of stem cell therapy. In the present study, we investigated the safety and efficacy of intra‐arterial administration of MSCs at different cell doses using a double‐toxin‐induced animal model of MSA.

## Materials and Methods

### Animal Study and Experimental Composition

To create a double toxin‐induced animal model of MSA, adult male or female Sprague Dawley rats (200–220 g) were injected with 6‐hydroydopamine (6‐OHDA, Sigma, St. Louis, MO, USA) and quinolinic acid (QA, Sigma) 16. 6‐OHDA (8 μg/4 μl) was injected by stereotaxic injection into the left medial forebrain bundle (A −2.2, L + 1.5 relative to bregma, V −8.0 relative to the dura, and with the tooth bar 4.5 mm above the interaural line). Three–four weeks after 6‐OHDA injection, a total of 150 nmol/4 µl QA was stereotaxically injected into two sites of the left striatum (A +1.4/+3.0, L +3.5/+3.0 relative to bregma, V −4.0 relative to the dura, and with the tooth bar 4.5 mm above the interaural line). All injections were made over 1 minute, and the needle was left in place for a further 2 minutes before being slowly withdrawn. The rats were randomly divided into three groups (*n* = 5; each group): (1) the control group; (2) the normal saline‐treated group; (3) the MSC‐treated group. The animal study was composed of two consecutive experiments; the first experiment was to test feasibility and distribution of intra‐arterially injected MSCs and the second experiment was to exam efficacy of different doses of MSCs. The animal work was approved by the Institutional Animal Care and Use Committees of Yonsei University.

### MSC Transplantation

One week after QA injection, MSCs were injected into the ICA or tail vein. Based on MSC concentration used by previous clinical trials 10, a concentration of 4 × 10^7^ cells/60 kg was selected as a standard dose of MSCs. The cell dose of human MSCs (hMSCs) used in the first experiment was a ×10 and ×50 of the standard dose of MSCs. Then, a ×0.2, ×2, and ×10 of the standard dose were used in the second experiment. For ICA injection, animals were anesthetized with 30% isoflurane. According to the previous experiment 14, the common carotid artery (CCA) was exposed and the external carotid artery and pterygopalatine artery were ligated. MSCs in 100 μl of solvent (Corestem, Inc., Seoul, Korea) were injected via a 33G microneedle into the CCA with preserved blood flow in the CCA and ICA for 30 seconds and bleeding was controlled by application of bioabsorbable Gelfoam (Pfizer, New York, NY, USA) at the injection site for 5 minutes. Pressure was maintained at the injection site until bleeding ceased and then fixed external carotid artery and pterygopalatine artery opened.

### Isolation of hMSCs

Bone marrow aspirates (10 ml) were obtained from the iliac crests of human donors. The mononuclear cell layer was isolated by Corestem, Inc. At passage 6, hMSCs were injected into rats via the CCA or tail vein.

### Fluorescence Activated Cell Sorting Analysis

The characterization of hMSCs was evaluated by fluorescence activated cell sorting (FACS) analysis. Briefly, cell suspensions were washed twice with phosphate buffered saline (PBS, Sigma) containing 0.1% bovine serum albumin (BSA, Sigma). For direct assays, 1 × 10^5^ cells/ml were incubated with fluorescein isothiocyanate (FITC)‐conjugated CD34 and CD45 (negative markers) as well as CD105 and anti‐human CD73 (positive markers) at 4°C for 30 minutes, and then washed twice with PBS containing 0.1% BSA. The cells were analyzed by cytometric analysis using an EPICS XL flow cytometer (Becton‐Dickinson, San Diego, CA, USA) with EXPO32 software.

### Behavioral Test

Three weeks after the 6‐OHDA lesion, apomorphine‐induced rotation was assessed (apomorphine hydrochloride, 0.5 mg/kg S.C. dissolved in sterile water; Sigma). Only animals showing a mean of 6 contralateral rotations/minutes over 30 minutes following the administration of apomorphine were retained. Behavioral test was repeated 2–3 weeks after striatal lesioning with QA as well as 1, 2, and 3 weeks after MSC transplantation.

### Tissue Preparation

For immunohistochemistry, rats were perfused with a saline solution containing 0.5% sodium nitrate and heparin (10 U/ml) and fixed with 4% paraformaldehyde dissolved in 0.1 M phosphate buffer (PB) (∼50 ml/rat) at 8 weeks after the first injection. Brains were removed from the skulls, post‐fixed overnight in buffered 4% paraformaldehyde at 4°C, and stored in a 30% sucrose solution for 1–2 days at 4°C until they sank. Coronal sections (30 μm) were obtained and stored in tissue stock solution (30% glycerol, 30% ethylene glycol, 30% three times distilled water, 10% 0.2 M PB) at 4°C until use.

### Immunohistochemistry

The coronal brain sections (4 μm thick) were rinsed twice in PBS and incubated in 0.2% Triton X‐100 for 30 minutes at room temperature (RT). They were rinsed three times and blocked with 0.5% BSA in ×1 PBS. After blocking, sections were incubated at 4°C overnight with the following primary antibodies: mouse anti‐tyrosine hydroxylase (TH; 1:2,000; Pel‐freez, Rogers, AR, USA), rabbit anti‐dopamine‐ and cAMP‐regulated neuronal phosphoprotein (DARPP‐32) (1:7,500; Abcam, Cambridge, MA, USA), rabbit anti‐Annexin V (1:500; Abcam, Cambridge, MA, USA), mouse anti‐NeuN (1:500; Abcam, Cambridge, MA, USA), or mouse anti‐nuclear matrix (NuMA; 1:100; Calbiochem, San Diego, CA, USA). Following overnight incubation, the brain sections were rinsed three times with 0.5% BSA in ×1 PBS (10 minutes/rinse) and incubated with the appropriate biotinylated secondary antibody and avidin–biotin complex (Elite Kit; Vector Laboratories, Burlingame, CA, USA) for 1 hour at RT. Bound antibodies were visualized by incubating the sections with 0.05% diaminobenzidine–HCl (DAB, Dako, carpinteria, CA, USA) and 0.003% hydrogen peroxide in 0.1 M PB. The brain sections were rinsed with 0.1 M PB for DAB inhibition. Immunostained cells were analyzed by bright‐field microscopy and viewed under a Zeiss LSM 780 confocal imaging system (Zeiss, Jena, Thuringia, Germany). To analyze the localizations of antigens in double‐stained samples, immunofluorescence images were created from the same tissue sections and merged using the Zeiss ZEN software (Zeiss).

### MSC Labeling for In Vivo Fluorescent Imaging

MSCs were labeled with NEO‐LIVE^TM^‐Magnoxide675 (Biterials, Seoul, South Korea), a fluorescent magnetic nanoparticle used for in vivo fluorescent imaging. NEO‐LIVE^TM^‐Magnoxide675 nanoparticles were prepared according to the manufacturer's protocol and MSCs were incubated for 24 hours with labeling solution containing NEO‐LIVE^TM^‐Magnoxide 675 at a concentration of 0.4 mg/ml in cultured media. Before animal injection, labeled MSCs were analyzed by fluorescence microscopy.

### Optical In Vivo Imaging System and Magnetic Resonance Imaging

For fluorescence images, animals were anesthetized with 30% isoflurane and images obtained using an Spectrum in vivo imaging system (IVIS; Caliper Lifesciences, Hopkinton, Massachusetts, USA) for data acquisition and analysis at 1 day, 3 days, 1 week, 2 weeks, 4 weeks, and 8 weeks after MSC transplantation. For magnetic resonance imaging (MRI), animals were anesthetized with 30% isoflurane, and images were obtained using a BioSpec 94/20 USR MRI (Bruker BioSpin, Ettlingen, Germany) for data acquisition and analysis 1 week after MSC transplantation. The in vivo imaging parameters were: (1) a three‐dimensional T2*‐weighted gradient echo sequence with repetition time (TR) = 2,500 ms and echo time (TE) = 24 ms for transverse sections of the brain, (2) a T2‐weighted two‐dimensional spin echo sequence with TR = 3,151 ms and TE = 24 ms. The MR angiography (MRA) scan was performed under a time of flight (TOF) sequence technique (TR = 18.0 ms, TE = 4.0 ms, flip angle (FA) = 80°, slice thickness = 0.35 mm, field of view (FOV) = 40 mm × 40 mm, and matrix = 256 × 256).

### Ultrasonic Blood Flow Measurement

For operative procedures, animals were anesthetized with 10% isoflurane in a mixture of 70% N_2_O and 30% O_2_. Anesthesia was maintained with 2% isoflurane. During operative procedures, body temperature was monitored continuously with a rectal probe and maintained at 37.0 ± 0.2°C using a homeothermic blanket control unit and a heating pad (Harvard Apparatus, Holliston, MA, USA). A midline cervical incision was made, and the left CCA was carefully dissected under a surgical microscope. An ultrasonic Doppler flow probe (MA0.7PSB; Transonic Instruments, Ithaca, NY, USA) was placed around midportion of the CCA. Carotid blood flow was obtained with a Transonic TS420 Blood Flow Meter (Transonic Instruments, Ithaca, NY, USA) and an iWorx IX‐304T data acquisition system (iWorx Systems, Inc., Dover, NH). Computer‐based analysis by iWorx Labscribe 2 software (version 2.045000) was performed to minimize any bias when assessing results. CCA baseline flow was measured for 3 minutes.

### Stereological Cell Counts

The total number of cells in the substantia nigra (SN), striatum, and frontal cortex was estimated using an optical fractionator and unbiased stereology of stained cells, as previously described 17. The sections used for counting covered the entire SN from the rostral tip of the pars compacta back to the caudal end of the pars reticulata. This generally yielded 8–9 sections in a series. Sampling was performed using the Olympus BX51 microscope (Olympus, Tokyo, Japan), and the SN was delineated at ×1.25 objective. A counting frame (60%, 35 frames, 650 μm^2^) was placed randomly on the first counting area and systematically moved though all counting areas until the entire delineated area was sampled. Actual counting was performed using a ×40 oil objective lens. Guard volumes (4 μm from the top and 4–6 from the bottom of the section) were excluded from both surfaces to avoid the problem of lost cap, and only the profiles that came into focus within the counting volume (with a depth of 10 μm) were counted. The total number of stained cells was calculated according to the optical fractionator formula. To measure striatal density, images of DARPP immunostaining in the striatum were captured. The numbers of DARPP‐positive cells per mm^2^ were measured using ImageGauge. For quantitative measure of apoptosis in the frontal cortex, it was defined as a percentage of Annexin V‐positive cells per total number of NeuN‐positive cells.

### Statistical Analysis

Comparisons between groups were made using Student's *t* tests (paired) or one‐way analysis of variance followed by a Dunnett post‐hoc tests. p Values less than .05 were considered statistically significant. Data were expressed as mean standard deviation. Statistical analyses were performed using commercially available software (version 10.0; SPSS, Inc., Chicago, IL).

## Results

### Characterization of hMSCs

**Figure 1:**
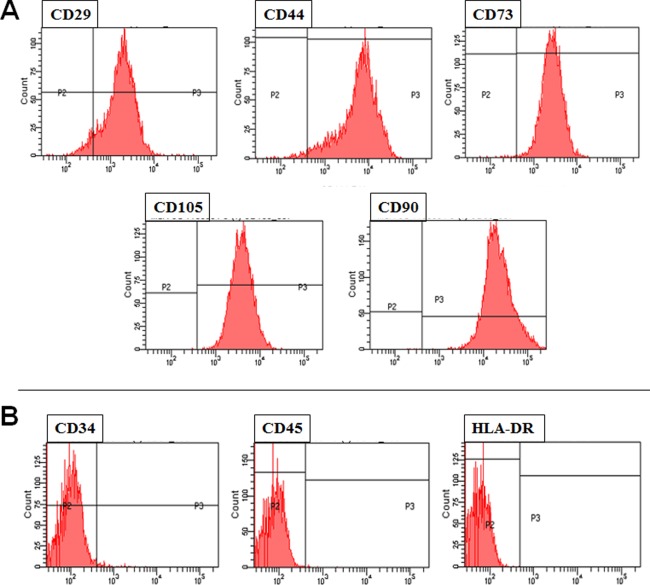
Flow cytometric analysis of human mesenchymal stem cells, showing positive markers **(A)** and negative markers **(B).**

FACS analysis confirmed that the hMSCs obtained for this study expressed CD29, CD44, CD73, CD105, and CD90, which are positive markers for hMSCs (Fig. 1A). Furthermore, hMSCs did not express CD34, CD 45, and HLA‐DR, which are negative markers for hMSCs (Fig. 1B).

### The First Experiment

#### Evaluation of ICA Flow and Cerebral Ischemia Following Intra‐Arterial Injection of MSCs

**Figure 2:**
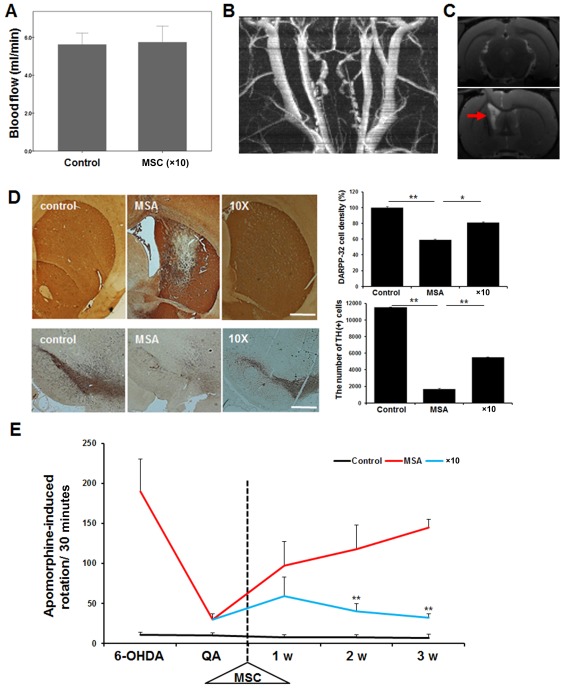
Evaluation of internal carotid artery (ICA) flow and cerebral ischemia following intra‐arterial injection of a ×10 of the standard. **(A)**: An ultrasonic flow meter showed that the ICA blood flow was well preserved in animals receiving MSCs. **(B)**: No occlusive lesion of the ICA was observed at a ×10 of the standard dose on magnetic resonance angiography. **(C)**: No hyperintense signals were observed in the brain parenchymal of animals receiving MSCs on T2‐weighted magnetic resonance imaging. Arrow indicates striatal lesion induced by 3‐nitroproprionic acid (3‐NP). **(D)**: Immunohistochemical analysis showed that MSC treatment significantly decreased the number of TH‐positive and DARPP‐32‐positive neurons in the striatum (upper panel) and the substantia nigra (lower panel) of double‐toxin treated animals (each group, *n* = 5). Scale bar represents 200 µm. **(E)**: Behavior performance in MSA animals receiving MSCs. The MSA animals receiving ×10 standard doses of MSCs showed significantly the number of rotations after apomorphine injection compared with placebo‐treated MSA animals (each group, *n* = 10). The data are presented as the means ± SE. *, *p* < .05; **, *p* < .01. Abbreviations: DARPP‐32, dopamine‐ and cAMP‐regulated neuronal phosphoprotein; MSA, multiple system atrophy; MSC, mesenchymal stem cell; 6‐OHDA, 6‐hydroydopamine; QA, quinolinic acid; TH, tyrosine hydroxylase.

To evaluate ICA patency that might be influenced by intra‐arterial administration of MSCs, an ultrasonic flow meter was used to measure blood flow through the ICA. The ICA blood flow was well preserved in animals receiving a ×10 of the standard dose of MSCs (Fig. 2A). In addition, to evaluate any delayed adverse effect of ICA occlusion, we performed MRI 4 weeks after MSC administration. No occlusive lesion of the ICA was observed at a ×10 of the standard dose (Fig. 2B). Using T2‐weighted MRI to detect ischemic brain lesions, no hyperintense signals were observed in the brain of animals receiving a ×10 of the standard dose (Fig. 2C). At the ×50 of the standard dose of MSCs, no animals survived more than 1 day after intra‐arterial MSC administration because MSCs were regurgitated from the injection site of the ICA with concomitant bleeding.

#### Histological and Behavioral Analysis

Immunohistochemical analysis revealed that MSA animals receiving double toxins showed a significant reduction of TH‐positive and DARPP‐32‐positive neurons in the SN and striatum compared with control animals (Fig. 2D). Compared with MSA animals receiving placebo, MSA animal receiving a ×10 of the MSC standard dose in MSA animals led to a significant attenuation of TH‐positive and DARPP‐32‐positive neurons in the SN and striatum (Fig. 2D). We did not perform immunohistochemical analysis in MSA animals receiving a ×50 of the standard dose of MSCs because of subject death from intra‐arterial MSC administration. On behavioral analysis, the number of rotations after apomorphine injection was significantly increased in double toxin‐induced MSA animals compared with control animals (Fig. 2E). However, MSA animals receiving a ×10 of the standard dose of MSCs exhibited a significant reduction in rotation behavior 1 week after MSC treatment compared with MSA animals receiving placebo (Fig. 2E).

### The Second Experiment

#### Evaluation of ICA Flow and Cerebral Ischemia Following Intra‐Arterial Injection of Different Doses of MSCs

**Figure 3:**
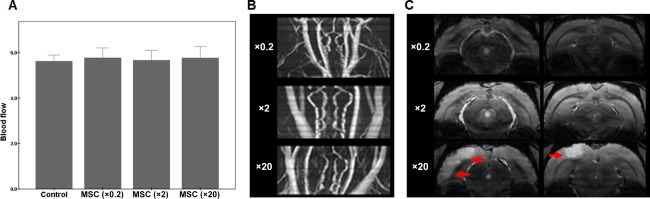
Evaluation of internal carotid artery (ICA) flow and cerebral ischemia following intra‐arterial injection of different doses of MSCs. **(A)**: An ultrasonic flow meter showed that the ICA blood flow was well preserved in animals receiving ×0.2, ×2, or ×20 of the standard dose. **(B)**: Magnetic Resonance‐angiography showed that no occlusive lesion of the ICA was observed at any of the MSC standard doses. **(C)**: T2*‐weighted magnetic resonance imaging revealed that multiple system atrophy animals receiving a ×20 of the standard dose of MSCs exhibited high signal intensity in the middle cerebral artery territory (arrows). Abbreviation: MSC, mesenchymal stem cell.

To evaluate efficacy of intra‐arterially injected MSCs, we chose ×0.2, ×2, or ×20 of the standard dose that was individual dose below ×10 of the standard dose or between ×10 and ×50 of the standard dose of MSCs. The ICA blood flow evaluated by an ultrasonic flow meter was well preserved in animals receiving the ×0.2, ×2, and ×20 of the standard doses of MSCs (Fig. 3A). In addition, no occlusive lesion of the ICA was observed at any of the MSC standard doses on MR‐angiography (Fig. 3B). On T2*‐weighted MRI, no hyperintense signals were observed in the brain parenchymal of animals receiving a ×0.2 and ×2 of the standard doses. However, MSA animals receiving a ×20 of the standard dose of MSCs exhibited high signal intensity in the middle cerebral artery territory (Fig. 3B) without hemorrhagic lesions.

#### Histological and Behavioral Analysis

**Figure 4:**
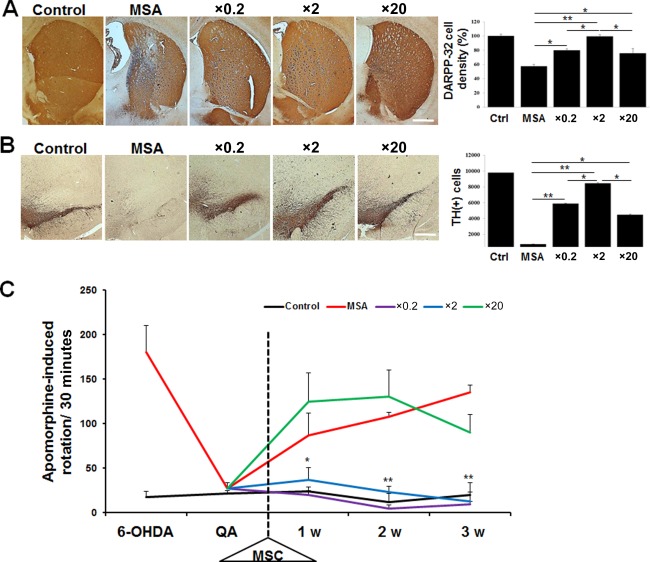
Histological and behavioral analysis following intra‐arterial injection of different doses of MSCs. **(A, B)**: Immunohistochemical analysis showed that MSC treatment with a ×0.2, ×2, or ×20 of the standard dose in MSA animals exhibited a significant increment of TH‐ and DARPP‐32‐positive neurons in the SN and striatum compared with MSA animals receiving placebo. A direct comparison revealed that prosurvival effect on TH‐positive and DARPP‐32‐positive neurons was higher in MSA animals receiving a ×2 of the standard dose compared with those receiving a ×0.2 or ×20 of the standard dose (each group, *n* = 5). Scale bar represents 200 µm. **(C)**: Behavioral analysis showed that MSA animals receiving MSCs at ×0.2 and ×2 concentrations of the standard dose exhibited a significant reduction in rotation behavior after apomorphine injection compared with MSA animals receiving placebo or ×20 of the standard dose of MSCs. (each group, *n* = 10). The data are presented as the means ± SE. *, *p* < .05; **, *p* < .01. Abbreviations: DARPP‐32, dopamine‐ and cAMP‐regulated neuronal phosphoprotein; MSA, multiple system atrophy; MSC, mesenchymal stem cell; 6‐OHDA, 6‐hydroydopamine; QA, quinolinic acid; TH, tyrosine hydroxylase.

Immunohistochemical analysis showed that MSC treatment with a ×0.2 or ×2 of the standard dose in MSA animals led to a significant increment of TH‐positive and DARPP‐32‐positive neurons in the SN and striatum compared with MSA animals receiving placebo (Fig. 4A, 4B). In direct comparison, the number of TH‐positive and DARPP‐32‐positive neurons was significantly higher in MSA animals receiving a ×2 of the standard dose compared with those receiving a ×0.2 of the standard dose (Fig. 4A, 4B). In MSA animals receiving a ×20 of the standard dose of MSCs, the number of TH‐positive and DARPP‐32‐positive neurons was significantly higher compared with those receiving placebo. However, the prosurvival effect on TH‐positive and DARPP‐32‐positive neurons in MSA animals receiving a ×20 of the standard dose was inferior to a ×2 of the standard dose (Fig. 4A, 4B). In addition, MSC treatment with a ×0.2, ×2, or ×20 of the standard dose in MSA animals decreased significantly Annexin V‐positive cells in the frontal cortex compared with MSA animals receiving placebo. Anti‐apoptotic effect was prominent in MSA animals receiving a ×2 of the standard dose compared with those receiving a ×0.2 or ×20 of the standard dose (Supporting Information Fig. S1). Behavioral analysis showed that MSA animals receiving MSCs at ×0.2 and ×2 concentrations of the standard dose exhibited a significant reduction in rotation behavior 1 week after MSC treatment compared with MSA animals receiving placebo (Fig. 4C). In a direct comparison, there were no significant differences in rotation behavior between the ×0.2 and ×2 of the standard dose of MSCs at each post‐treatment time (Fig. 4C). However, the number of rotations after apomorphine injection in MSA animals receiving a ×20 of the standard dose did not differ significantly compared with placebo‐treated MSA animals (Fig. 4C).

#### Intracerebral Concentrations of MSCs

**Figure 5:**
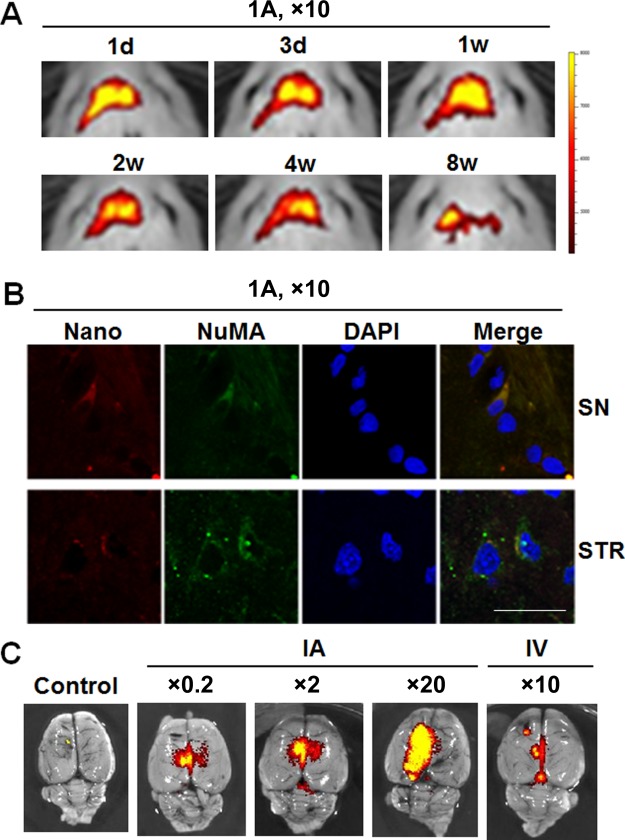
Intracerebral concentrations of MSCs. **(A)**: In vivo imaging system analysis showing serial changes of fluorescent signal intensity in the brain following intra‐arterial (IA) injection of a ×10 standard dose. **(B)**: NuMA‐positive MSCs comerged with nanoparticle in the SN and STR 8 weeks after intra‐arterial administration. Scale bar represents 10 µm **(C)**: Distribution patterns of MSCs in the brain depending on MSC dose. Fluorescent signal intensity in the brain 1 day after IA administration exhibited an incremental tendency as the concentration of MSCs was increased. Following intravenous administration of a ×10 of the standard, fluorescent signal intensity density in the brain was lower than intra‐arterial injection of a ×0.2 of the standard dose. Abbreviations: SN, substantia nigra; STR, striatum.

First, we examined how long MSCs would survive in the brain following intra‐arterial injection of MSCs at a ×10 standard dose. IVIS analysis showed that the distribution of MSCs in the brain gradually decreased over time, and 8 weeks after MSC injection, fluorescent signal intensity in the brain was markedly attenuated (Fig. 5A). Additionally, NuMA‐positive MSCs comerged with nanoparticle were identified in the SN and striatum 8 weeks after intra‐arterial administration (Fig. 5B). Next, we evaluated distribution patterns of MSCs in the brain depending on MSC dose. As expected, the distribution of MSCs in the brain 1 week after intra‐arterial administration had an incremental tendency as the concentration of MSCs was increased (Fig. 5C). Finally, we used intravenous injection of MSCs to evaluate whether intracerebral concentrations of MSCs might differ depending on delivery routes. When MSCs were administrated at a ×10 of the standard dose via the tail vein, fluorescent signal intensity density in the brain was even lower than intra‐arterial injection of a ×0.2 of the standard dose (Fig. 5C).

## Discussion

The present study revealed that if their concentration is appropriate, intra‐arterial injection of MSCs is safe and exerts a neuroprotective effect on striatal and nigral neurons with a coincidental improvement in motor behavior. However, in addition to the fatal effect of an extremely high MSC dose, intra‐arterial injection of a higher dose of MSCs led to the development of ischemic lesions and thus attenuated improvement of behavioral performance. Furthermore, IVIS analysis showed that intra‐arterial injection of MSCs increases cellular density in the brain compared with intravenous injection. These data indicate that intra‐arterial administration of MSCs is feasible and efficacious with respect to neuronal viability and behavioral performance in double toxin‐induced MSA models.

In terms of the pathogenesis of MSA, some clues to understanding the mechanisms of MSA have been suggested. Using MSA transgenic mice, studies have suggested that mitochondrial inhibitors or proteasome inhibitors might trigger or aggravate MSA pathologies 18, 19. Taken together, dysfunction in cellular clearing systems and neuroinflammation may cause further deterioration of these neurodegenerative processes 20. Until now, many clinical trials have been performed to uncover the efficacy of various treatments in patients with MSA; however, these trials have failed to provide ample evidence of neuroprotection or had limited efficacy in MSA patients 21. These results imply that if a certain molecular candidate is to achieve a neuroprotective effect in MSA patients, this candidate may need to fully prevent cell death cascades in every pathogenic step.

With the idea that MSCs may have pleiotropic effects against the neurodegenerative microenvironment, the present study used a double‐toxin‐induced animal model of MSA to examine the feasibility and efficacy of intra‐arterial administration of various doses of MSCs to extend its clinical application to patients with MSA. In the present study, a concentration of MSCs that was used in previous clinical trials (4 × 10^7^ MSCs) was selected as a standard concentration. We found that MSA animals only receiving ×0.2 or ×2 of the standard dose of MSCs exhibited behavioral improvement relative to the placebo‐treated group, whereas prosurvival effects of MSCs in immunohistochemical analysis were evident in MSA animals receiving ×0.2, ×2, or ×20 of the standard dose. In a comparative analysis among MSC treatment subgroups, behavioral analysis showed no significant difference in motor performance between MSA animals receiving ×0.2 and ×2 of the standard dose; however, immunohistochemical analysis demonstrated that prosurvival effects on striato‐nigral neurons were more prominent in MSA animals receiving the ×2 of the standard dose compared with those receiving a ×0.2 or ×20 of the standard dose. Therefore, combining behavioral and histological analyses, the present data indicate that a ×2 of the standard dose of MSCs may be the most efficacious dose of intra‐arterial injection in MSA animals.

We also found that intra‐arterial administration of higher doses of MSCs led to in situ occlusion of the injected artery or ischemic insults in the distal cerebral arterial circulation. Specifically, when MSA animals were injected with a ×50 of the standard dose, the cells were regurgitated from the injection site in the ICA probably due to arterial occlusion, which led to higher mortality. In addition, intra‐arterial injection with a ×20 of the standard dose was associated with ischemic lesions in the distal MCA territory. This may explain why MSA animals receiving the ×20 of the standard dose of MSCs did not show a significant improvement in behavioral performance and neuronal survival compared with those receiving a ×0.2 or ×2 of the standard dose. In terms of ischemic complication associated with intra‐arterial injection of MSCs, cell dose, and patency of arterial flow are important factors in determining the development of ischemic insults, even though the complication rate appears to vary depending on the experimental design of intra‐arterial injection 14, 15, 22. Additionally, our previous clinical studies demonstrate that the frequency of ischemic insults is comparable between MSC‐treated and placebo‐treated groups, suggesting that ischemic lesions seem to be a consequence of the angiographic procedure rather than intra‐arterial MSC injection. Accordingly, the present study provides further in vivo evidence that if arterial flow is patent during intra‐arterial administration, the cellular density of MSCs around a standard dose is quite safe and free from ischemic insults in addition to providing neuroprotective efficacy. However, because we did not determine the infusion volume or infusion velocity as a contributing factor to the development of cerebral ischemia 23. A further study would be required to resolve safety issues related with intra‐arterial injection of MSCs.

IVIS analysis showed that intra‐arterial injection of MSCs markedly increased migration of MSCs into the brain compared with intravenous injection. MSCs characteristically migrate toward injured brain areas in various animal models of ischemia and Parkinson's disease, possibly in response to signals that are upregulated under injury conditions 24, 25. SDF‐1 is widely expressed in the brain, including the cortex, cerebellum, basal ganglia, and SN pars compacta 25. Damage in the SN and striatum induced by 1‐methyl‐4‐phenyl‐1,2,3,6‐tetrahydropyridine and 3‐NP may increase the expression of SDF‐1 and CXCR4, leading to recruitment of MSCs to these regions. As expected, the present study showed that the distribution of intra‐arterially injected MSCs in the brain had an incremental tendency as the concentration of MSCs was increased. Together, we directly compared the tendency of homing effects between intra‐arterial and intravenous routes by analyzing IVIS. The number of homing MSCs after intravenous administration even at the ×10 of the standard dose of MSCs was much lower than intra‐arterial injection of a ×0.2 of the standard dose. As these migrated cells may contribute to modulation of the microenvironmental cascade of the neurodegenerative process in the brain, the intra‐arterial route would be by far superior to the intravenous route in terms of bioavailability of homing MSCs.

Finally, we found that when a ×10 dose of MSCs was administrated via the intra‐arterial route, the concentration of MSCs in the brain gradually decreased over time, and 8 weeks after MSC injection, the cellular signal intensity in the brain was markedly attenuated. Considering the survival time of MSCs, this may raise the possibility of the need for additional MSC administrations to bolster homing MSCs. Similarly, our previous clinical trial demonstrates that the neuroprotective effect of MSCs tend to fade away around day 240 after the first intra‐arterial MSC injection, implying repeated administration of MSCs may be needed to maintain their neuroprotective properties 10. However, further studies determining the most appropriate time interval of repetitive MSC administrations are required for clinical application in patients with MSA.

## Conclusion

Our study has provided important details that intra‐arterial administration of MSCs is feasible and efficient administration route, resulting in improvement of neuronal viability and behavioral performance in double toxin‐induced MSA models. The present data may advance clinical applications of intra‐arterial route of MSC administration in patients with MSA.

## Author Contributions

P.H.L.: designed and supervised the entire study, interpreted data analysis, provided financial support, and wrote manuscript; O.S.H., K.H.N., and K.D.Y.: performed experiments and analyzed the data; K.H.S. and K.K.S.: provided cells, performed experiments, and discussed and analyzed the data.

## Disclosure of Potential Conflicts of Interest

The authors indicated no potential conflicts of interest.

## Supporting information

Supporting Information
