# Supplementary material for: Feasibility and Efficacy of Intra‐Arterial Administration of Mesenchymal Stem Cells in an Animal Model of Double Toxin‐Induced Multiple System Atrophy
Source: Stem Cells Transl Med. 2017 Mar 13;6(5):1424–33. doi: 10.1002/sctm.16-0438 (PMC5442709; doi:10.1002/sctm.16-0438)

**Supplementary Figure 1.** Immunostaining of Annexin V following intraarterial injection of different doses of MSCs. MSC treatment with a 0.2×, 2×, or 20× of the standard dose in MSA animals decreased significantly Annexin V-positive cells in the frontal cortex compared with MSA animals receiving placebo (left). Quantification analysis showed that anti-apoptotic effect was prominent in MSA animals receiving a 2× of the standard dose compared with those receiving a 0.2× or 20 ×of the standard dose (right). Scale bar represents 20 µm. *p < 0.05; **p < 0.01.


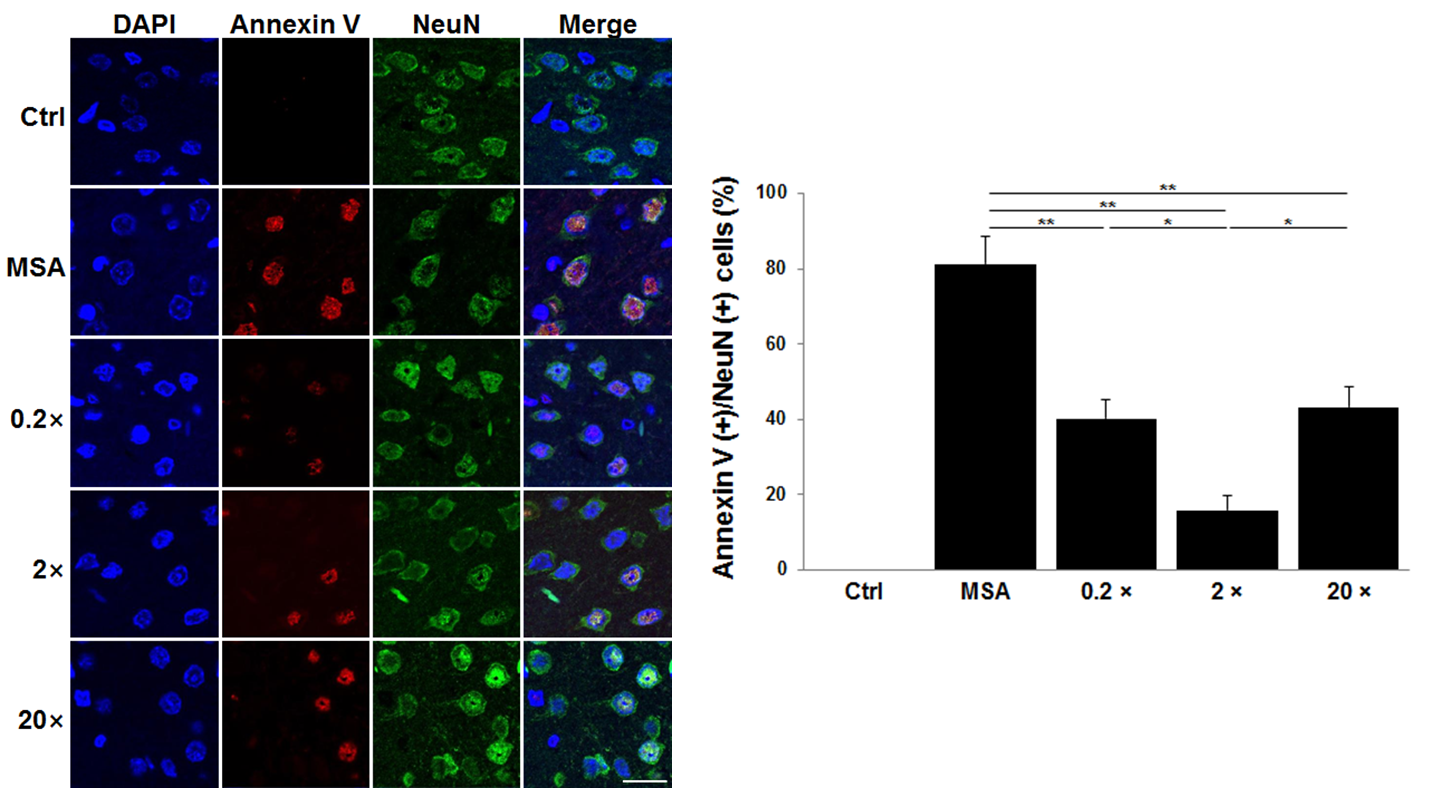

Supplement: Supplementary file 1 — Supporting Information [file SCT3-6-1424-s001.doc]
